# Visualizing the *in-vivo application* of zinc in sensitive skin using reflectance confocal microscopy

**DOI:** 10.1038/s41598-021-87346-0

**Published:** 2021-04-08

**Authors:** Hye-Jin Ahn, Hae Jin Kim, Hyein Ham, Ji Hwoon Baek, Young Lee, Mahin Alamgir, Babar Rao, Min Kyung Shin

**Affiliations:** 1 Department of Medicine, Graduate School, Kyung Hee University, Seoul, South Korea; 2 Department of Dermatology, Kyung Hee University Medical Center, Seoul, South Korea; 3 Dermapro Skin Research Center, DERMAPRO Ltd, Seoul, South Korea; 4 Department of Dermatology, School of Medicine, Chungnam National University, Daejeon, South Korea; 5 Department of Dermatology, Rutgers Robert Wood Johnson Medical School, Somerset, NJ USA; 6 Department of Dermatology, Weill Cornell Medical Center, New York, NY USA; 7 Department of Dermatology, College of Medicine, Kyung Hee University, # Kyung HeeDae Ro 23, Dongdaemun-gu, Seoul, 02447 South Korea

**Keywords:** Medical research, Signs and symptoms

## Abstract

Findings obtained on objective assessments to evaluate sensitive skin do not correlate well with the symptomatology. We utilized reflectance confocal microscopy (RCM) to compare transepidermal *application* of zinc in sensitive and non-sensitive skin. Thirty-six subjects participated in this study. They were divided into groups based on lactic acid sting test (LAST):‘stinger’ and ‘non-stinger’; transepidermal water loss (TEWL) measurements; and sensitivity self-assessments: ‘sensitive’ and ‘non-sensitive’. RCM images were taken to visualize *transepidermal application* of topically-applied zinc. The intensity of zinc reflectance at different depths was measured by ImageJ software. Based on LAST scores, the ‘stinger’ group showed significantly higher *reflectance* of zinc at 8 µm (stratum corneum) [face (*P* < 0.001), forearm (*P* = 0.004)], and at 80–104 µm (dermo-epidermal junction layer) on the face. High-TEWL group showed increased zinc *reflectance* at 8–24 µm (tight junction layer, *P* < 0.001). There were no significant differences amongst subjects self-reporting ‘sensitive’ and ‘non-sensitive’ skin. RCM demonstrates that in sensitive skin, there is deeper and higher *reflectance* of zinc at multiple depths. Structural differences are also visualized. We suggest that RCM is a useful tool for evaluating skin barrier integrity.

## Introduction

There is no widely accepted definition of sensitive skin (SS). However, SS has recently been characterized as a syndrome defined by the occurrence of unpleasant subjective symptoms (stinging, burning, pain, pruritus, and tingling sensations) in response to stimuli that usually should not provoke such sensations^1, 2^.

Impaired skin barrier function has been suggested to be the underlying cause of SS^3–5^, and its association with atopic conditions^6–8^. Several tests have been used to evaluate SS, such as the lactic acid sting test (LAST), thermal sensitivity test, capsaicin test, and histamine test^9^. Objective assessments include measuring the transepidermal water loss (TEWL) after applying sodium lauryl sulfate, dimethylsulfoxide provocation test, colorimetry, corneometry, and laser Doppler velocimetry^9^. However, objective findings do not necessarily correlate with the subjective symptoms^9^. SS is believed to be caused by an increase in the permeability of the stratum corneum (SC), leading to greater transdermal penetration^10^.

Reflectance confocal microscopy (RCM) is a noninvasive method for visualizing the dynamic status of the epidermal and upper dermal structures. In a study using RCM to compare atopic dermatitis and normal skin, the healthy skin showed a uniform arrangement of a polygonal mesh of furrows, which was not seen in the atopic skin^11^. To our knowledge, there have been few studies evaluating the use of RCM to analyze SS in otherwise disease-free skin. We performed in vivo RCM analysis of the skin to evaluate the integrity of the epidermal barrier.

## Results

### *Zinc reflectance* on RCM in groups based on LAST scores

**Figure 1:**
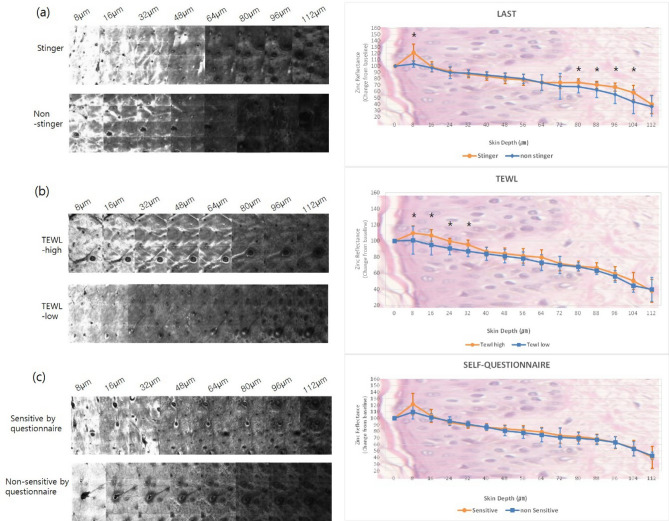
RCM images and the zinc reflectance values on the face. The reflectance intensity of zinc at 8 µm in the ‘stinger’ group was higher compared to the non-stinger group (*P* < 0.001). There were significant differences between the two groups from 80 to 104 µm (*P* = 0.048 at 80 µm, *P* = 0.037 at 88 µm, *P* = 0.008 at 96 µm, *P* = 0.042 at 104 µm) (**a**). The high-TEWL group showed greater zinc *reflectance* from depths of 8 μm to 32 μm on the face (*P* = 0.011 at 8 μm, *P* < 0.001 at 16–32 μm) (**b**). There were no significant differences in zinc *reflectance* on the face in subjects self-identifying as having ‘sensitive’ or ‘non-sensitive’ skin (**c**). (*P < 0.05).

**Figure 2:**
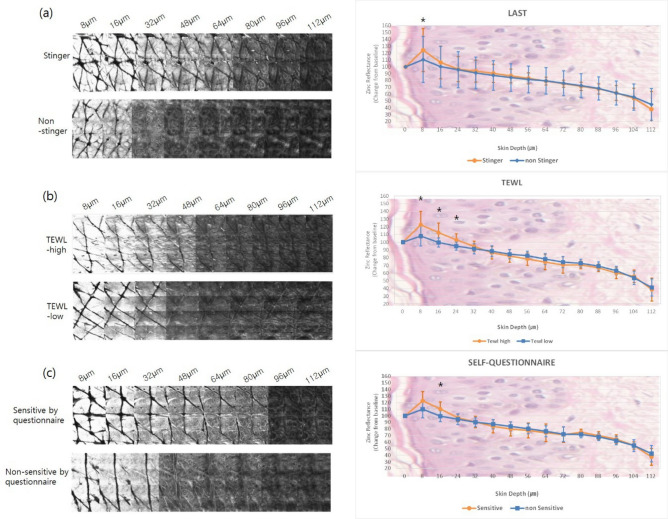
RCM images and the zinc reflectance values on the forearm. The reflectance intensity of zinc at 8 µm in the ‘stinger’ group was greater than the in ‘non-stinger’ group (*P* = 0.004) (**a**). The high-TEWL group showed greater zinc *reflectance* from 8 μm to 24 μm depth on the forearm (*P* = 0.009 at 8 μm, *P* < 0.001 at 16 μm, *P* = 0.001 at 24 μm) (**b**). There was a significant difference at the 16 μm depth (*P* = 0.003) in subjects self-identifying as having ‘sensitive’ skin compared to ‘non-sensitive’ skin (**c**). (*P < 0.05).

Based on the LAST scores, subjects were divided into a ‘stinger’ group (n = 18 subjects; 6 males and 12 females), and a ‘non-stinger group’ (n = 18 subjects; 9 males and 9 females). In the stinger group, *zinc application* on the face increased progressively from a depth of 0 to 8 µm (Fig. 1a). Subsequently, *the reflectance* decreased and almost leveled off at depths of 24 to 64 µm. There was a statistically significant difference between the ‘stinger’ and ‘non-stinger’ groups at a depth of 8 µm and in the deeper layers at depths of 80–104 µm (Supplementary Table S1). Zinc application on the forearm was significantly different only at a depth of 8 µm (Fig. 2a, Supplementary Table S1).

RCM images and the zinc reflectance values on the face. The reflectance intensity of zinc at 8 µm in the ‘stinger’ group was higher than that in the non-stinger group (*P* < 0.001). There were significant differences between the two groups from 80 to 104 µm (*P* = 0.048 at 80 µm, *P* = 0.037 at 88 µm, *P* = 0.008 at 96 µm, *P* = 0.042 at 104 µm) (a). The high-TEWL group showed greater *zinc reflectance* from depths of 8 μm to 32 μm on the face (*P* = 0.011 at 8 μm, *P* < 0.001 at 16–32 μm) (b). There were no significant differences in *zinc reflectance* on the face in the subjects who self-assessed themselves as having ‘sensitive’ or ‘non-sensitive’ skin (c). (*P < 0.05).

RCM images and the zinc reflectance values on the forearm. The reflectance intensity of zinc at 8 µm in the ‘stinger’ group was greater than that in the ‘non-stinger’ group (*P* = 0.004) (a). The high-TEWL group showed greater *zinc reflectance* from 8 to 24 μm depth on the forearm (*P* = 0.009 at 8 μm, *P* < 0.001 at 16 μm, *P* = 0.001 at 24 μm) (b). There was a significant difference at the 16 μm depth (*P* = 0.003) in subjects who self-assessed themselves as having ‘sensitive’ skin compared to those who self-assessed themselves as having ‘non-sensitive’ skin (c). (*P < 0.05).

### TEWL measurements in the ‘stinger’ and ‘non-stinger’ groups

There was no difference in the average TEWL measurements between the ‘stinger’ and ‘non-stinger’ groups. The average TEWL on the face was 17.4 gm^−2^ h^−1^ in the ‘stinger’ group and 17.8 gm^−2^ h^−1^ in the ‘non-stinger’ group (*P* = 0.81). The average TEWL on the forearm in the ‘stinger’ group was 9.3 gm^−2^ h^−1^ and it was 8.4 gm^−2^ h^−1^ in the ‘non-stinger’ group (*P* = 0.37) (Supplementary Table S2).

### Zinc reflectance on RCM in groups based on TEWL measurements

For evaluating the reflectance of zinc on the face, subjects were categorized as high-TEWL (n = 11; 8 males and 3 females) or low-TEWL (n = 25; 7 males and 18 females). Similarly, for evaluating the reflectance of zinc on the forearm, the subjects were categorized as high-TEWL (n = 20, 10 males and 10 females) and low-TEWL (n = 16, 5 males and 11 females).

On the face, there were statistically significant differences in the zinc reflectance between the high-TEWL and low-TEWL groups at depths of 8–32 µm (Fig. 1b, Supplementary Table S1). Similarly, on the forearm, RCM showed a significant difference in zinc reflectance at depths of 8 to 24 µm (Fig. 2b, Supplementary Table S1).

### Zinc reflectance on RCM based on self-assessed skin sensitivity questionnaire

Based on the self-assessment, 26 subjects (10 males and 16 females) were grouped into the ‘sensitive’ group and 10 subjects into the ‘non-sensitive’ group (5 males and 5 females). There were no subjects with co-existing dermatologic disorders. Among the ‘sensitive’ group, 11 subjects were in the ‘stinger’ group (based on the results of LAST) and 15 were in the ‘non-stinger’ group. Among the ‘non-sensitive’ group, 7 subjects were in the ‘stinger’ group and 3 subjects were in the ‘non-stinger’ group. Thus, more subjects made a subjective self-assessment of having SS than the number actually demonstrated through the results of LAST scores.

Among the ‘sensitive’ group, zinc reflectance on the face was slightly higher than that in the ‘non-sensitive’ group at depths from 8 to 16 µm. However, these differences were not significant (Fig. 1c, Supplementary Table S1). On the forearm, higher zinc reflectance was visualized in the ‘sensitive’ group at depths of 8 to 16 µm (Fig. 2c), and this difference was statistically significant at 16 µm (Supplementary Table S1).

### Dermoscopic images and mosaic RCM images of the groups based on LAST

The mean scores of dermoscopic images in the ‘stinger’ group were higher (11.53) than in the ‘non-stinger’ group (10.25) (Supplementary Figure S3). Among the evaluation criteria, telangiectasia (‘stinger’: 2.24, ‘non-stinger’: 1.63) and dyschromia (‘stinger’: 2.71, ‘non-stinger’: 2.19) showed the most significant difference between the two groups.

**Figure 3:**
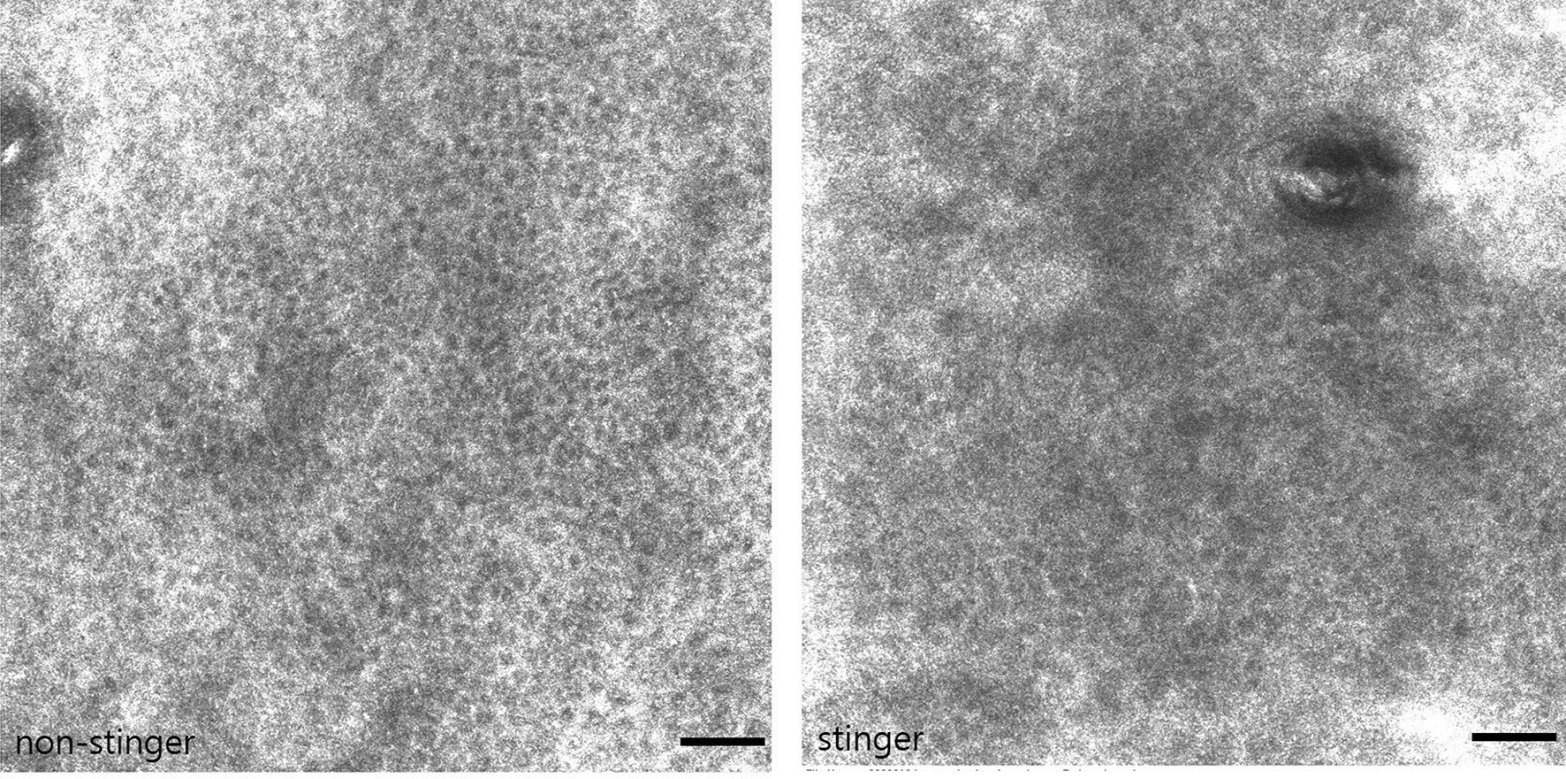
RCM images of the spinous layer on the face. A regular honeycomb pattern in a ‘non-stinger’ subject and irregular honeycomb pattern in a ‘stinger’ subject at 24 μm depth (Scale bar: 50 μm).

In mosaic RCM images, the mean score of the honeycomb structures in the ‘stinger’ group (1.06) was slightly higher than that in the ‘non-stinger’ group (0.81) (Fig. 3). In the images of 500 × 500 μm^2^ on RCM, follicular structures were visualized in 6 subjects in the ‘stinger’ group and 12 subjects in the ‘non-stinger’ group. However, there was no apparent perifollicular *zinc reflectance* in either group.

RCM images of the spinous layer on the face. A regular honeycomb pattern in a ‘non-stinger’ subject and an irregular honeycomb pattern in a ‘stinger’ subject at 24 μm depth (Scale bar: 50 μm).

## Discussion

The pathophysiology underlying the SS is not completely understood^10^. A systematic study showed that the density of intraepidermal nerve fibers, especially peptidergic C-fibers, is lower in the SS; however, a decrease in epidermal thickness or an increase in the epidermal inflammatory markers was not seen^12^. Previous studies have suggested that the mechanisms underlying SS are neither immunologic nor allergic^12–14^; however, there is an increase in the permeability of the SC, which leads to greater penetration of substances and more water loss^10^.

Several reports have investigated the relationship between the skin barrier and SS. There is an inverse relationship between the corneocyte size/SC thickness and skin permeability, and changes in this mechanical barrier lead to abnormal skin penetration by the irritants^15^. A study reported that in those with SS, the quantity of ceramides in the SC on the face was significantly lower^16, 17^, which implies an epidermal barrier dysfunction. However, a recent study investigating the molecular composition of the skin barrier in SS using confocal Raman microspectroscopy found no differences in the SC thickness and the content of water or natural moisturizing factor between the sensitive and non-sensitive skin (NSS)^18^. The authors concluded that the poor correlation between the findings obtained through biophysical techniques and observed alterations in the molecular composition of the skin barrier, emphasizing the need for the use of objective tools for in vivo skin barrier analysis^18^.

In this study, zinc sulfate was used to provide higher reflectivity for RCM images. Zinc, especially zinc oxide nanoparticles that are commonly used in sunscreen formulations. A study of human skin penetration of topical nano zinc oxide showed zinc oxide nonopartiacle failed to penetrate into the viable epidermis of intact human skin, with no detectable levels. In contrast, it was found in the upper layer of the viable epidermis of barrier-impaired skin^19^.

Considering the depth at which a significant decrease in *zinc reflectance* occurred across all the groups in our study, we suggest that tight junctions, thought to be located in the outermost layer of the stratum granulosum in humans^20–22^, are present at a depth of about 16–24 µm. Given that in the ‘high-TEWL’ group, an overall higher *zinc reflectance* through all the epidermal layers was noted, we postulate that in the skin with high TEWL, concomitant defects in both SC and tight junctions exist. In contrast, the ‘stinger’ and the self-perceived ‘sensitive’ group showed only an SC defect, with undamaged tight junctions in the upper epidermal layer. This result is consistent with the increased TEWL seen with defects in tight-junction proteins^23^.

Measurement of TEWL has been used to aid in the diagnosis of SS^10^. However, a recent study showed that there is no statistical difference between SS and NSS on TEWL measurements^5^. This is in accordance with our findings, which showed no statistical difference in TEWL values between the ‘stinger’ and ‘non-stinger’ groups (Supplementary Table S2), despite the ‘stinger’ group and ‘high-TEWL’ group showing greater zinc reflectance. Another difference between the ‘stinger’ group and the ‘high-TEWL’ group was that the ‘stinger’ group showed a higher reflectance than the ‘non-stinger’ group at depths of 80–104 µm on the facial skin. These results suggest that the facial skin in the ‘stinger’ group is associated with an impairment of both the SC barrier and the dermo-epidermal junction (DEJ). Using confocal laser scanning microscopy in ex vivo, the bulk of free nerve endings in tissue sections is visualized just below the DEJ^24^. Although a further study is necessary, impairment of the DEJ could be attributed to the sting sensation experienced during LAST. Meanwhile, there are several previous studies that reported epidermal thicknesses measured by RCM. These epidermal thicknesses were 48.28 μm (± 13.0) in the volar forearm in a younger age group^25^ and 45.07 μm (± 10.64) in the face in an elderly population^26^. Our result of the DEJ was depths of 80–104 µm, which was a deeper and wider range compared to the previous studies. Wurm et al*.* measured the minimal epidermal thickness^25^. Cinotti et al*.* measured from the top of the SC^26^. However, we measured depths starting at the base of the SC. Additionally, we suggest the DEJ’s wide range appeared to reflect the DEJ's hills-and-valleys topography^27^.

A possible explanation for the difference in reflectance between the face and forearm could be due to regional differences in the size of the corneocyte. Several studies have reported smaller corneocytes in the facial areas relative to those in the forearm^28, 29^. The SC of facial skin is thinner and undergoes a more rapid turnover^30^. The unique functional characteristics of the facial skin maintain better hydration of the skin surface, but lead to a relatively weak barrier function^30^.

Some researchers have proposed an association between SS and dermatological diseases such as atopic dermatitis, rosacea, and psoriasis, and several studies using RCM have shown the loss of honeycomb structures and the absence of bright papillary rims in inflammatory skin diseases^11, 31, 32^. At a depth of 24 µm, there was an irregular honeycomb pattern in the ‘stinger’ group on RCM, and we hypothesize that this structural irregularity suggests an existing subclinical inflammatory state.

This is the first study that used RCM to demonstrate in vivo differences in the *zinc reflectance* of the skin barrier in SS compared to NSS. Our results also demonstrate that the ‘stinger’ group shows greater *reflectance* in the SC layer. We further found that findings in the ‘high-TEWL’ group do not necessarily match those of the ‘stinger’ group; however, the higher *zinc reflectance* demonstrated on RCM implies a skin barrier dysfunction in both groups. These differences were not seen among the groups who self-assessed their skin as ‘sensitive’. Overall, self-assessments of SS correlated least with the extent of *zinc reflectance* as visualized on RCM, and thus might be less accurate in predicting actual epidermal barrier defects. In conclusion, RCM demonstrates that in SS, there is a deeper and higher *reflectance* of zinc at multiple depths. Structural differences were also visualized. We conclude that RCM is a useful tool for evaluating integrity of the skin barrier.

## Materials and methods

### Subjects

Thirty-six subjects (15 males, 21 females) aged 20–58 years (mean age, 41.25 years) participated in this study. The DERMAPRO LTD. Institutional Review Board approved the study (220777-A-N-02-DICN19040). Informed consent was obtained from all the subjects before enrollment. All the procedures were performed in accordance with relevant guidelines. The subjects were divided into groups based on the LAST scores (‘stinger’ group n = 18, ‘non-stinger’ group n = 18), TEWL measurements on the face (high-TEWL group n = 11, low-TEWL group n = 25), TEWL measurements on the forearm (high-TEWL group n = 20, low-TEWL group n = 16), and scores of the self-assessed skin sensitivity questionnaire (sensitive group n = 26, non-sensitive group n = 10).

### Lactic acid sting test

Each subject’s face was washed and stabilized under constant temperature (22 ± 2 °C) and relative humidity (50 ± 5%) for 30 min. The LAST was conducted according to the Frosch and Kligman method^33^ (“5% LAST”); 50 mL of lactic acid (5%) and distilled water (DW) were added dropwise to a filter paper (1 × 1 cm^2^), which was placed on the nasolabial folds. At 0, 2.5, 5, and 8 min, any stinging, burning, or itching reported by the subjects was recorded on a 4-point scale (0, no stinging; 1, slight stinging; 2, moderate stinging; 3, severe stinging). Subjects with scores of 0.2 or higher were selected as a ‘stinger’ [Score = (Total lactic acid value-Total DW value)/12 (4-point scale × 3 items)].

### Transepidermal water loss measurements

The test was conducted in an area away from the wind and direct sunlight. The room temperature was 20–24 °C, and the relative humidity was 45–55%. The probe of a Tewameter TM 300 (Courage & Khazaka Electronic GmbH, Cologne, Germany) was placed on the face and distal left forearm. The measurement was performed for 30 to 45 s. We defined a high-TEWL on the face as at least 18 gm-2 h-1 and a high-TEWL on the forearm as at least 8 gm-2 h-1^34^.

### Self-assessed skin sensitivity questionnaire

Participants answered 18 questions about their skin sensitivity and recorded symptoms of skin discomfort, stinging, redness, or dryness following exogenous and endogenous triggers (i.e., toiletries, metals, heat, food, alcohol, emotions). A history of dermatologic disorders such as dermatitis, acne, or rosacea was also obtained. Each question was scored on a scale of 1 to 4 points. A total score of 30 or more was considered as having 'sensitive skin.'

### Zinc reflectance on RCM

**Figure 4:**
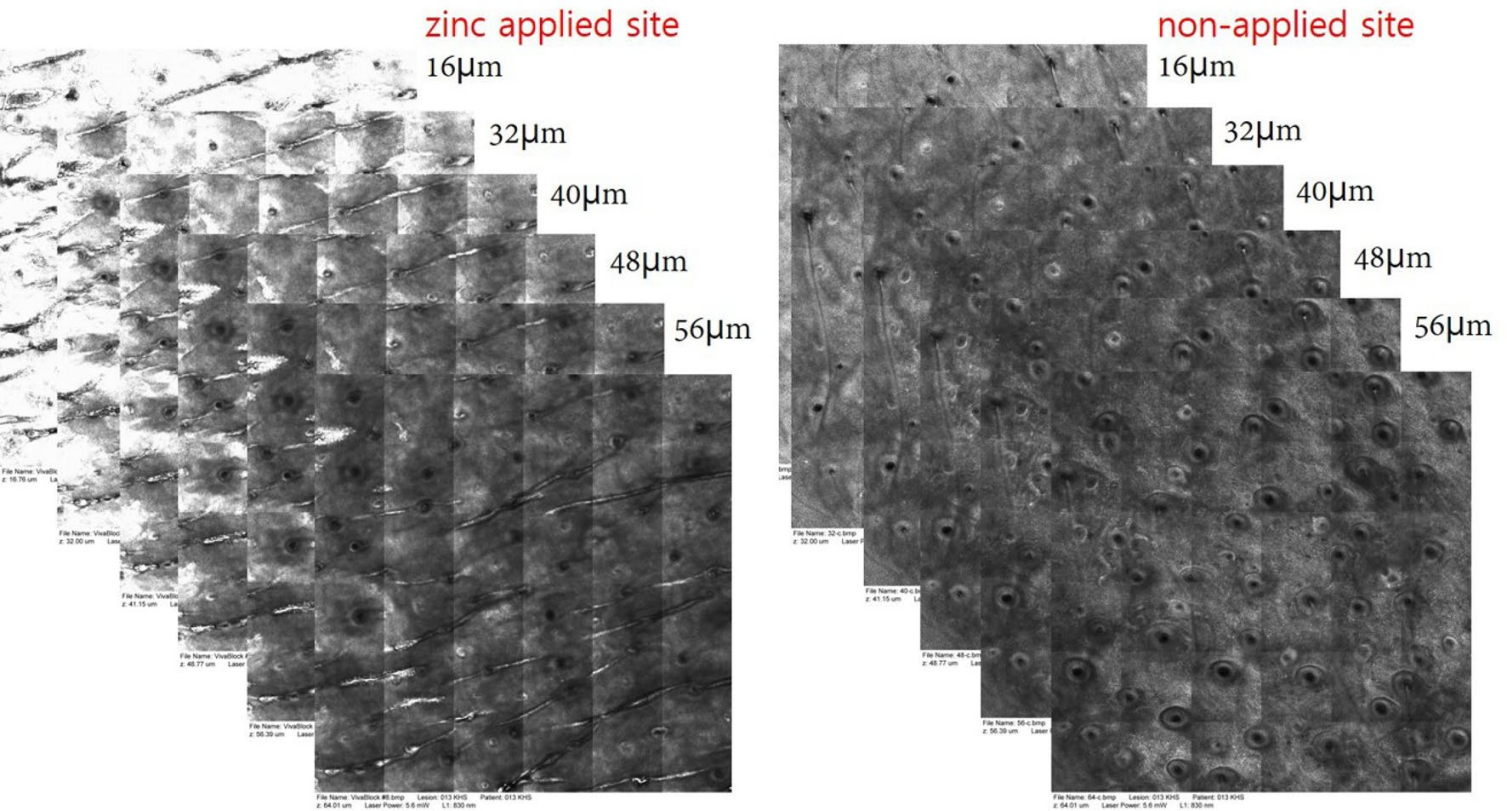
RCM images after zinc application. After zinc application, the facial skin showed higher reflectance than the non-applied zinc facial skin at different depths.

We captured the real-time scan sequence using RCM (VivaScope1500, Lucid Inc., Rochester, NY) after application of 2 mL (approximately 1 mg/cm^2^) zinc (zinc sulfate hydrate 4.4 mg/mL; 179.47 g/mol, Samjin Pharm, South Korea) on the face and forearm. RCM images were taken 60 min after topical application. Zinc provided high reflectance on the RCM images (Fig. 4). We used laser power at 5.8–6.0 W and horizontal composite images of 4 × 4 mm^2^ (confocal mosaics) for the analysis. We used ImageJ software to measure the intensity of zinc reflectance. Since the reflectance of RCM according to the melanin content varies between each individual, we corrected the zinc reflectance by subtracting the baseline reflectance value of each skin layer. Baseline reflectance was obtained from the surrounding skin without zinc application. We considered the deep layer of the SC as the most superficial epidermal layer (0 µm). We set the zinc reflectance intensity value as 100 at an epidermal depth of 0 µm, and the remaining values were calculated as relative values. Finally, the corrected intensity values at each skin depth were plotted on graphs.

RCM images after zinc application. After zinc application, the facial skin showed higher reflectance at different depths than that of the areas on which zinc was not applied.

### Analysis of dermoscopic and mosaic images of the groups based on LAST

We analyzed the dermoscopic images taken from ‘stinger’ and ‘non-stinger’ groups (based on the LAST scores) using a digital macro camera (Vivacam; Lucid Inc., Rochester, NY), which was linked to the RCM. Dermoscopic images were evaluated for erythema, telangiectasias, eccrine duct orifice hyperpigmentation, perifollicular hyperkeratosis, and dyschromia. Scores were assigned to each feature (1: none, 2: weak, 3: strong). We also captured the RCM images showing the honeycomb structures and perifollicular zinc *reflectance* at a depth of 24 µm in the ‘stinger’ and ‘non-stinger’ groups. The quality of the honeycomb structure was determined as: 0, regular structure; 1, slightly irregular; and 2, very irregular.

### Statistical analysis

We performed a Wilcoxon rank-sum test to identify significant differences in the zinc *application reflectance*, and a t-test was used to compare the TEWL between the ‘stinger’ and ‘non-stinger’ groups (based on LAST scores). Data were analyzed using SPSS for Windows (version 23.0, SPSS Inc., Chicago, IL). For all statistical tests, a P-value < 0.05 was considered statistically significant.

## Supplementary Information

Supplementary Information 1.

Supplementary Information 2.

Supplementary Information 3.
