# Supplementary material for: Visualizing the in-vivo application of zinc in sensitive skin using reflectance confocal microscopy
Source: Sci Rep. 2021 Apr 8;11:7738. doi: 10.1038/s41598-021-87346-0 (PMC8032733; doi:10.1038/s41598-021-87346-0)
Supplement: Supplementary file 1 — Supplementary Information 1. [file 41598_2021_87346_MOESM1_ESM.docx]

Visualizing the *in-vivo* application of zinc in sensitive skin using reflectance confocal microscopy

Hye-Jin Ahn^1,2^, Hae Jin Kim^2^, Hyein Ham^3^, Ji Hwoon Baek^3^, Young Lee^4,5^, Mahin Alamgir ^5^, Babar Rao^5,6^_,_ Min Kyung Shin^1,2^

^1^Department of Medicine, Graduate School, Kyung Hee University, Seoul, South Korea , ^2^Department of Dermatology, Kyung Hee university medical center, Seoul, South Korea

^3^Dermapro Skin Research Center, DERMAPRO Ltd., Seoul, South Korea, ^4^Department of Dermatology, School of Medicine, Chungnam National University, Daejeon, South Korea, ^5^Department of Dermatology, Rutgers Robert Wood Johnson Medical School, Somerset, New Jersey, USA, ^6^Department of Dermatology, Weill Cornell Medical Center, New York, New York, USA

Word Count of abstract: 183

Word count of text: 2940

Number of references: 35

Figures: 4

Supplementary Material: 3

Correspondence: Min Kyung Shin, MD, PhD.

Associate Professor, Department of Dermatology, College of Medicine, Kyung Hee University

# Kyung HeeDae Ro 23, Dongdaemun-gu, Seoul, 02447, Republic of Korea

E-mail: haddal@hanmail.net, Telephone number: 82-2-958-8300, Fax: 82-2-969-6538

**Supplementary 1. Zinc reflectance by RCM of groups.**

a) Statics analysis by Wilcoxon rank-sum test showed significant differences in the intensity value of confocal reflectance based on lactic acid sting test (LAST) at a depth of 8 μm and from 80μm to 104 μm on the face. There was a significant difference in intensity value of confocal reflectance based on TEWL from depths of 8 μm to 32 μm on the face but no significant difference on the face based on self- questionnaire.

| **Face** | **SC**  **(8** **μm)** | **TJ**  **(16~24** **μm)** | **SS**  **(32** **μm)** | **SB**  **(40~80** **μm)** | **Upper dermis**  **(88~104** **μm)** |
| --- | --- | --- | --- | --- | --- |
| LAST  Stinger vs Non stinger | <0.001 | - | - | 0.048 | 0.037 |
| TEWL  Higher vs Lower | 0.011 | <0.001 | <0.001 | - | - |
| Self-questionnaire  Sensitive vs non sensitive | - | - | - | - | - |

*LAST: lactic acid sting test, TEWL: transepidermal water loss, SC:stratum corneum, TJ: tight junction, SS: stratum spinosum, SB:stratum basale

b) Statics analysis by Wilcoxon rank-sum test showed a significant difference in the intensity value of confocal reflectance based on lactic acid sting test at a depth of 8 μm on the arm. Intensity value of confocal reflectance based on TEWL was significantly different from a depth of 8 μm to 24 μm on the arm. Intensity value of confocal reflectance based on self- questionnaire showed a significant difference at a depth of 16 μm on the arm.

| **Arm** | **SC**  **(8** **μm)** | **TJ**  **(16~24** **μm)** | **SB**  **(40~80** **μm)** | **Upper dermis**  **(88~104** **μm)** |
| --- | --- | --- | --- | --- |
| LAST  Stinger vs Non stinger | 0.004 | - | - | - |
| TEWL  Higher vs Lower | 0.009 | <0.001 | - | - |
| Self-questionnaire  Sensitive vs non sensitive | - | 0.003 | - | - |

*LAST: lactic acid sting test, TEWL: transepidermal water loss, SC:stratum corneum, TJ: tight junction, SB:stratum basale
