# Supplementary material for: Visualizing the in-vivo application of zinc in sensitive skin using reflectance confocal microscopy
Source: Sci Rep. 2021 Apr 8;11:7738. doi: 10.1038/s41598-021-87346-0 (PMC8032733; doi:10.1038/s41598-021-87346-0)
Supplement: Supplementary file 2 — Supplementary Information 2. [file 41598_2021_87346_MOESM2_ESM.docx]

Visualizing the *in-vivo* application of zinc in sensitive skin using reflectance confocal microscopy

Hye-Jin Ahn^1,2^, Hae Jin Kim^2^, Hyein Ham^3^, Ji Hwoon Baek^3^, Young Lee^4,5^, Mahin Alamgir ^5^, Babar Rao^5,6^_,_ Min Kyung Shin^1,2^

^1^Department of Medicine, Graduate School, Kyung Hee University, Seoul, South Korea , ^2^Department of Dermatology, Kyung Hee university medical center, Seoul, South Korea

^3^Dermapro Skin Research Center, DERMAPRO Ltd., Seoul, South Korea, ^4^Department of Dermatology, School of Medicine, Chungnam National University, Daejeon, South Korea, ^5^Department of Dermatology, Rutgers Robert Wood Johnson Medical School, Somerset, New Jersey, USA, ^6^Department of Dermatology, Weill Cornell Medical Center, New York, New York, USA

Word Count of abstract: 183

Word count of text: 2940

Number of references: 35

Figures: 4

Supplementary Material: 3

Correspondence: Min Kyung Shin, MD, PhD.

Associate Professor, Department of Dermatology, College of Medicine, Kyung Hee University

# Kyung HeeDae Ro 23, Dongdaemun-gu, Seoul, 02447, Republic of Korea

E-mail: haddal@hanmail.net, Telephone number: 82-2-958-8300, Fax: 82-2-969-6538

**Supplementary 2. Transepidermal water loss of all subjects.**

| **Stinger (face)** | | | | **Non-stinger (face)** | | |
| --- | --- | --- | --- | --- | --- | --- |
| No. | **Age** | **Sex** | **TEWL** | **Age** | **Sex** | **TEWL** |
| 1 | 48 | M | 11.5 | 20 | F | 10.1 |
| 2 | 52 | F | 12.0 | 47 | F | 12.1 |
| 3 | 50 | F | 13.1 | 50 | F | 13.2 |
| 4 | 23 | F | 13.1 | 49 | F | 14.1 |
| 5 | 43 | F | 14.3 | 47 | F | 14.1 |
| 6 | 23 | F | 14.3 | 53 | M | 14.2 |
| 7 | 38 | F | 14.7 | 36 | F | 15.2 |
| 8 | 46 | F | 14.7 | 39 | F | 16.0 |
| 9 | 38 | F | 15.9 | 38 | F | 16.6 |
| 10 | 41 | F | 16.1 | 32 | M | 16.9 |
| 11 | 28 | M | 16.1 | 29 | M | 18.3 |
| 12 | 31 | F | 18.0 | 58 | M | 18.6 |
| 13 | 52 | M | 18.0 | 47 | F | 19.9 |
| 14 | 47 | F | 21.3 | 48 | M | 20.1 |
| 15 | 36 | F | 21.4 | 45 | M | 21.3 |
| 16 | 38 | M | 22.3 | 36 | M | 21.1 |
| 17 | 56 | M | 23.1 | 34 | M | 23.7 |
| 18 | 43 | M | 32.6 | 44 | M | 35.0 |

| Stinger (mean) | Non-stinger (mean) | P-value |
| --- | --- | --- |
| 17.4 | 17.8 | 0.8063 |

| **Stinger (arm)** | | | | **Non-stinger (arm)** | | |
| --- | --- | --- | --- | --- | --- | --- |
| No. | **Age** | **Sex** | **TEWL** | **Age** | **Sex** | **TEWL** |
| 1 | 41 | F | 4.5 | 20 | F | 4.2 |
| 2 | 48 | M | 5.2 | 58 | M | 4.2 |
| 3 | 50 | F | 5.8 | 29 | M | 6.1 |
| 4 | 52 | F | 6.2 | 49 | F | 6.2 |
| 5 | 56 | M | 7.2 | 50 | F | 6.5 |
| 6 | 38 | F | 7.9 | 47 | F | 6.9 |
| 7 | 43 | F | 8.9 | 48 | M | 7.0 |
| 8 | 23 | F | 9.1 | 36 | F | 7.1 |
| 9 | 38 | F | 9.1 | 47 | F | 8.8 |
| 10 | 47 | F | 9.1 | 39 | F | 9.3 |
| 11 | 38 | M | 9.2 | 38 | F | 9.3 |
| 12 | 31 | F | 9.7 | 32 | M | 9.6 |
| 13 | 52 | M | 10.1 | 45 | M | 9.6 |
| 14 | 46 | F | 11.1 | 34 | M | 9.8 |
| 15 | 28 | M | 12.0 | 47 | F | 10.0 |
| 16 | 43 | M | 12.7 | 53 | M | 10.2 |
| 17 | 36 | F | 14.7 | 36 | M | 10.9 |
| 18 | 23 | F | 15.7 | 44 | M | 16.3 |

| Stinger (mean) | Non-stinger (mean) | P-value |
| --- | --- | --- |
| 9.3 | 8.4 | 0.3676 |
