# Supplementary material for: Visualizing the in-vivo application of zinc in sensitive skin using reflectance confocal microscopy
Source: Sci Rep. 2021 Apr 8;11:7738. doi: 10.1038/s41598-021-87346-0 (PMC8032733; doi:10.1038/s41598-021-87346-0)

Visualizing the *in-vivo* application of zinc in sensitive skin using reflectance confocal microscopy

Hye-Jin Ahn^1,2^, Hae Jin Kim^2^, Hyein Ham^3^, Ji Hwoon Baek^3^, Young Lee^4,5^, Mahin Alamgir ^5^, Babar Rao^5,6^_,_ Min Kyung Shin^1,2^

^1^Department of Medicine, Graduate School, Kyung Hee University, Seoul, South Korea , ^2^Department of Dermatology, Kyung Hee university medical center, Seoul, South Korea

^3^Dermapro Skin Research Center, DERMAPRO Ltd., Seoul, South Korea, ^4^Department of Dermatology, School of Medicine, Chungnam National University, Daejeon, South Korea, ^5^Department of Dermatology, Rutgers Robert Wood Johnson Medical School, Somerset, New Jersey, USA, ^6^Department of Dermatology, Weill Cornell Medical Center, New York, New York, USA

Word Count of abstract: 183

Word count of text: 2940

Number of references: 35

Figures: 4

Supplementary Material: 3

Correspondence: Min Kyung Shin, MD, PhD.

Associate Professor, Department of Dermatology, College of Medicine, Kyung Hee University

# Kyung HeeDae Ro 23, Dongdaemun-gu, Seoul, 02447, Republic of Korea

E-mail: haddal@hanmail.net, Telephone number: 82-2-958-8300, Fax: 82-2-969-6538

**Supplementary Figure S3. Dermoscopic images score.**

Examples of dermoscopic images (Digital macro camera connected to RCM (Vivacam; Lucid Inc., Rochester, NY)) on face of ‘stinger’ (a) and ‘non-stinger’ (b). The mean scores of the ‘stinger’ group were higher than the ‘non-stinger’ group (c).


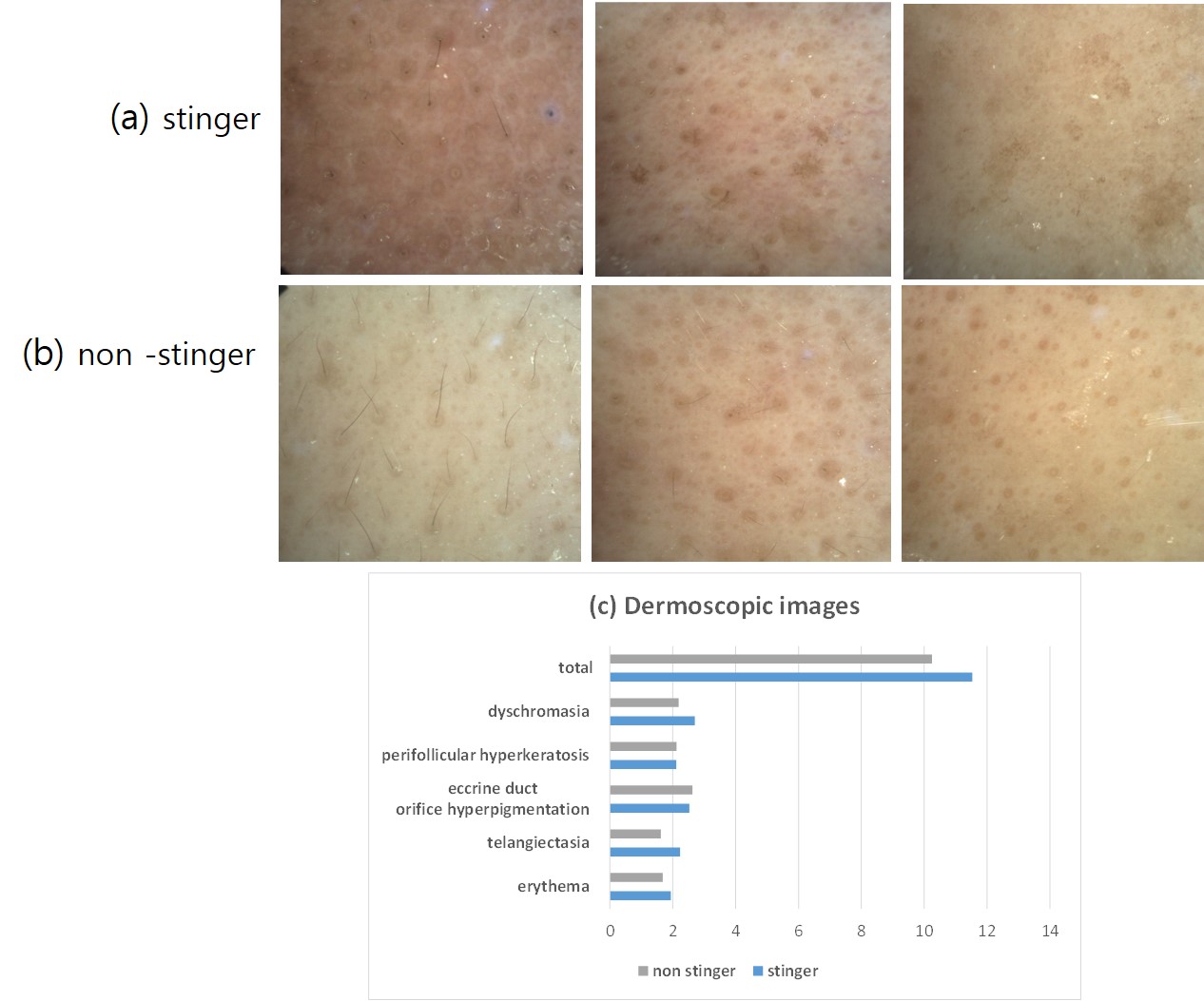

Supplement: Supplementary file 3 — Supplementary Information 3. [file 41598_2021_87346_MOESM3_ESM.docx]
